# Reduction of Adipose Tissue Formation by the Controlled Release of BMP-2 Using a Hydroxyapatite-Coated Collagen Carrier System for Sinus-Augmentation/Extraction-Socket Grafting

**DOI:** 10.3390/ma8115411

**Published:** 2015-11-11

**Authors:** Jung-Seok Lee, Tae-Wan Kim, Soyon Park, Byung-Soo Kim, Gun-Il Im, Kyoo-Sung Cho, Chang-Sung Kim

**Affiliations:** 1 Department of Periodontology, Research Institute for Periodontal Regeneration, College of Dentistry, Yonsei University, Seoul 03722, Korea; cooldds@gmail.com (J.-S.L.); ikale2@naver.com (T.-W.K.); kscho@yuhs.ac (K.-S.C.); 2 Department of Applied Life Science, BK21 PLUS Project, College of Dentistry, Yonsei University, Seoul 03722, Korea; soyonp923@gmail.com; 3 School of Chemical and Biological Engineering, Seoul National University, Seoul 08826, Korea; byungskim@snu.ac.kr; 4 Department of Orthopaedic Surgery, Ilsan Hospital, Dongguk University, Goyang 10326, Korea; gunil@dumc.or.kr

**Keywords:** bone morphogenetic protein, collagen, hydroxyapatite, drug delivery system, bone regeneration

## Abstract

The effects of hydroxyapatite (HA)-coating onto collagen carriers for application of recombinant human bone morphogenetic protein 2 (rhBMP-2) on cell differentiation *in vitro*, and on *in vivo* healing patterns after sinus-augmentation and alveolar socket-grafting were evaluated. *In vitro* induction of osteogenic/adipogenic differentiation was compared between the culture media with rhBMP-2 solution and with the released rhBMP-2 from the control collagen and from the HA-coated collagen. Demineralized bovine bone and collagen/HA-coated collagen were grafted with/without rhBMP-2 in sinus-augmentation and tooth-extraction-socket models. Adipogenic induction by rhBMP-2 released from HA-coated collagen was significantly reduced compared to collagen. In the sinus-augmentation model, sites that received rhBMP-2 exhibited large amounts of vascular tissue formation at two weeks and increased adipose tissue formation at eight weeks; this could be significantly reduced by using HA-coated collagen as a carrier for rhBMP-2. In extraction-socket grafting, dimensional reduction of alveolar ridge was significantly decreased at sites received rhBMP-2 compared to control sites, but adipose tissue was increased within the regenerated socket area. In conclusion, HA-coated collagen carrier for Escherichia coli-derived rhBMP-2 (ErhBMP-2) may reduce *in vitro* induction of adipogenic differentiation and *in vivo* adipose bone marrow tissue formation in bone tissue engineering by ErhBMP-2.

## 1. Introduction

The clinical applications of recombinant human bone morphogenetic protein (rhBMP-2) have been studied widely in bone tissue engineering since the development of protein-recombination technology [1]. Based on the vast amount of scientific evidence available from previous research, including *in vitro*/*in vivo* preclinical experiments and clinical studies [2,3,4,5], clinical use of rhBMP-2 has been approved by the Food and Drug Administration (FDA), although its use is restricted to limited indications: nonunion of long bones and spinal fusion in the field of orthopedics, and maxillary sinus and alveolar bone augmentation in the dental field [6,7,8].

In the field of dentistry, however, several other recent studies have raised questions regarding the rationale for using rhBMP-2 in sinus augmentation. Although it was expected that rhBMP-2 would enhance the bone quality in regenerated bone tissue, Kao *et al.* reported decreased bone quality in a histologic study (randomized clinical trial) of sinus augmentation with rhBMP-2 compared to the conventional technique [9]. Some also expected that rhBMP-2 would promote healing processes in bone regeneration, and reduce time to prosthetic loading. However, a recent preclinical study using rabbit sinus model revealed limited formation of mineralized tissue in the central area of the augmentation at sites received bone graft materials accompanied by rhBMP-2 [10]. These findings suggest that instead of promoting the early bone-healing processes within sinus augmentation, rhBMP-2 switched the direction of those processes.

Tissue responses to rhBMP-2 may be influenced by the characteristics or environment of the recipient site, including the type of affected cells, the presence of other growth factors from adjacent tissues, or the characteristics of the experimental subject. The defect type may also be an affecting factor. In the aforementioned study [10], the peculiar healing pattern was observed in a sinus-augmentation model, and it might not be found in other preclinical models such as ectopic subcutaneous/intramuscular models, long-bone defects, calvarial defects, and other augmentation models. Other studies suggest that the concentration/dose of applied rhBMP-2 is a critical factor in reducing the incidence of unwanted effects such as adipose tissue (AT) formation [11] or cystic changes [12]. The application of rhBMP-2 in tissue engineering enables the concentration of the protein at the defect site to be significantly higher than would be available in the normal physiologic state, especially with the initial burst release from the carrier system. Thus, control of the release from carriers could be a solution for the successful clinical application of rhBMP-2.

One representative rhBMP-2 carrier system is absorbable collagen sponge (ACS), which is the only carrier with FDA approval, and many other controlled-release carrier systems have been researched. Hydroxyapatite (HA)-coated collagen was recently introduced in an attempt to improve the weakness of the ACS carrier system by enabling controlled release and strengthening its mechanical stability. The aim of this biomaterial was to mimic the physicochemical properties of bone; it has been suggested that the negatively charged sites of the HA coating interact electrostatically with the positive charge of the amino acids in BMP-2 [13]. Yang *et al.* previously demonstrated the controlled release profile of rhBMP-2 from an HA-coated collagen carrier system and enhanced osteoinduction by Escherichia coli-derived rhBMP-2 (ErhBMP-2) for calvarial defect healing compared to conventional carrier of ACS [14]. They also suggested that AT formation in the early healing processes could be reduced by decreasing the initial availability of rhBMP-2 via the use of a controlled-release carrier system.

The aim of the present study was to determine whether using HA-coated collagen as a carrier for rhBMP-2 enhances the bony regeneration in two different types of defects, mimicking the limited clinical indications for which the FDA has approved its use. The specific objectives were twofold: (1) to determine the effects of an HA-coated collagen carrying rhBMP-2 on cell differentiation *in vitro*; and (2) to determine the *in vivo* healing pattern in the sinus-augmentation and alveolar socket-grafting models following implantation of rhBMP-2 with the HA-coated collagen carrier system.

## 2. Materials and Methods

### 2.1. Preparation of the HA-Coated Collagen Carrier

The HA-coated collagen carrier used in this study was provided by Professor Byung-Soo Kim from Seoul National University, and was produced with standardized protocol as described previously [14]. Briefly, commercially available type I ACS (HeliCOTE; Integra Life Sciences, York, PA, USA) was coated with HA by immersing/incubating in a simulated body fluid (SBF) solution, comprised reagent-grade NaCl (40.017 g/L), NaHCO_3_ (1.775 g/L), KCl (1.125 g/L), K_2_HPO_4_∙3H_2_O (1.155 g/L), MgCl_2_∙6H_2_O (1.555 g/L), CaCl_2_ (1.46 g/L), Na_2_SO_4_ (0.36 g/L), and (CH_2_OH)_3_CNH_2_ (30.285 g/L) dissolved in distilled water [15]. The collagen scaffolds were then carefully washed five times with distilled water, and lyophilized for 1 day. ACS used as a default carrier in all experiments of this study was same as the one that was used for aforementioned HA-coating protocol.

### 2.2. In Vitro Assay for Cell Differentiation Induced by the rhBMP-2 Released from Its Carriers

#### 2.2.1. Preincubation in the Cell-Growth Medium

The rhBMP-2 used in this study was provided by the research institute of Cowellmedi (Pusan, Korea). The rhBMP-2 was expressed in *Escherichia coli* (ErhBMP-2), as described previously [16]. Four types of cell culture medium were prepared for the experimental groups, according to the type of carrier and loading (or not) of ErhBMP-2 (*n* = 3 per group):

- Negative control group: standard culture medium.
- Positive control group: standard culture medium containing ErhBMP-2 (1 μg/mL).
- C/BMP-2 group: standard culture medium in which ErhBMP-2-soaked ACS was placed during the planned releasing periods, after presoaking with ErhBMP-2 (1 μg/mL) for 30 min. (C: absorbable collagen sponge)
- HA/BMP-2 group: standard culture medium with the same conditions as for the C/BMP-2 group, except that HA-coated collagen was used as the carrier instead of ACS.

Collagen and HA-coated collagen were cut into standardized circular sheets (3 mm thick and 8 mm in diameter) using a tissue punch. The culture media with ErhBMP-2 solution, rhBMP-2-loaded ACS, and rhBMP-2-loaded HA-coated collagen were preincubated for 3, 7 and 14 days to allow release of ErhBMP-2.

#### 2.2.2. Induction of Osteogenic and Adipogenic Differentiation

Cell differentiation (osteogenic and adipogenic) assays were performed to evaluate the bioactivity of the released ErhBMP-2 at three different releasing periods: determining responses to differentiative induction of cells stimulated by released ErhBMP-2 in each period. Human gingival fibroblasts (hGFs) were used for both cell differentiation assays. Gingival tissues from one patient receiving dental implant treatment were collected and hGFs were isolated. The experimental protocol was approved by the Ethics Committee of the College of Dentistry, Yonsei University (IRB No. 2-2010-0032), and written informed consent was obtained before enrollment to the study. Cells at 4th passage were seeded into six-well plates at a density of 1 × 10^5^ cells/well, and cultured in each medium for 5 days until they reached subconfluence. The culture medium was then replaced by the specific culture medium for osteogenic differentiation. After 4 weeks of induction, the cells were stained with Alizarin Red. For adipogenic differentiation, the cells were subjected to three cycles of adipogenic induction/maintenance, starting at confluence. Each cycle involved treating the cells with adipogenic induction medium for 3 days followed by adipogenic maintenance medium for the next 3 days. The adipogenic differentiation media were purchased from Lonza (Lonza Walkersville, Walkersville, MD, USA). At the end of 21 days of culture, mature adipocytes were detected by Oil Red O staining. The total areas of mineralized nodule formation and lipid vacuoles were measured using an automated image-analysis system (Image-Pro Plus, Media Cybernetics, Rockville, MD, USA).

### 2.3. In Vivo Assay for Tissue Regeneration Using Two Types of Animal Model

#### 2.3.1. Experimental Animals

Twenty male New Zealand white rabbits weighing 2.5–3.0 kg were selected for the rabbit sinus-augmentation model, and five male beagle dogs aged approximately 15 months and weighing 10–15 kg were used for the extraction-socket model. The animal selection, management, surgical protocol, and preparation were approved by the Institutional Animal Care and Use Committee of Yonsei Medical Center, Seoul, Korea.

#### 2.3.2. Study Design and Surgical Procedures

The same experimental design of group categorization was applied in both *in vivo* assays; however, deproteinized bovine bone mineral (DBBM) was added as a space-maintaining biomaterial in *in vivo* experiments for mimicking clinical situations. ACS and HA-coated collagen were minced into standardized, small pieces (2 mm × 2 mm) and mixed with DBBM at a proportion of 30 pieces of collagen per 150 mg of DBBM. At sites receiving ErhBMP-2, 30 pieces of both control and HA-coated collagen were soaked in 0.3 mL of ErhBMP-2 at a concentration of 0.25 mg/mL for 30 min, and then mixed with 150 mg of DBBM. The control and experimental groups were categorized as follows:

- Sham control group: sham surgery sites with no graft (included only in the extraction-socket grafting experiment).
- DBBM group: experimental sites received DBBM only.
- DBBM/C group: experimental sites received DBBM and ACS.
- DBBM/C/BMP-2 group: experimental sites received DBBM and ErhBMP-2-loaded ACS.
- DBBM/HA group: experimental sites received DBBM and HA-coated collagen.
- DBBM/HA/BMP-2 group: experimental sites received DBBM and ErhBMP-2-loaded HA-coated collagen.

Sinus-Augmentation Experiment: Surgical procedures for sinus augmentation were performed with the protocol as described previously; [10] briefly, bilateral/standardized/circular windows were prepared on roofs of maxillary sinus using a trephine bur. The trephined bony disk was carefully removed, and sinus membrane was then elevated. Forty sinuses in twenty rabbits were first divided into two groups according to the prescribed healing period: 2 and 8 weeks. Twenty sinuses in each group were assigned randomly to one of the 5 aforementioned experimental groups by randomly picking a sealed envelope inside which there was predesigned group information (*n* = 4 in 10 groups of 5 experimental conditions and 2 observational periods). Sham surgery control group was excluded in this experiment, due to its impracticality in this model; the space-maintaining grafting biomaterial (DBBM) is an indispensable factor in sinus augmentation. The prepared mixtures of DBBM (150 mg) with/without control collagen/HA-coated collagen and/or rhBMP-2 were implanted into the sinuses. The animals were sacrificed after 2 or 8 weeks postoperatively by anesthetic overdose.

Extraction-Socket Grafting Experiment: Unilateral mandibular second, third, and fourth premolars were hemisectioned, the six roots were gently extracted using a forcep. The sites were rotationally divided into one control group and five treatment groups for an even distribution (*n* = 5 in each group), and grafted with mixture of biomaterials up to the ridge crest according to group allocation. Primary closure was achieved by advancing and suturing the periosteal flaps. After 8 weeks of healing, the dogs were sacrificed by anesthetic overdose.

#### 2.3.3. Radiographic Analysis Using Micro-Computed Tomographic Images

Block sections including the augmented area and surrounding tissues were cut and fixed in 10% buffered formalin for 10 days. The fixed block specimens were scanned using micro-computed tomography (SkyScan 1072, SkyScan, Aartselaar, Belgium) at a resolution of 35 mm (100 kV, 100 μA). The scanned data sets were processed in digital imaging and communications in medicine (DICOM) format and reconstructed with computer-based software (On-Demand 3D, Cybermed, Seoul, Korea). In all sectioned planes, the total augmented areas were identified by color coding and traced manually using the software program, and the overall topography of experimental sites was reconstructed.

Sinus-augmentation experiment: The overall dimensional topography of the rabbit sinuses and supporting bone was visualized in a reconstructed image. The maximum augmented height (AH) was measured on the images of coronal sections. Among the serial sections, one image of each site was chosen by considering the highest peak in the reconstructed view based on linear measurements. The total augmented volume (AV) was calculated by integrating the color-coded data from all micro-computed tomographic images.

Extraction-socket grafting experiment: The central-most sagittal plane for each socket site was superimposed onto the same plane of a pristine tooth in the contralateral mandible, with references to the inferior and lingual borders of alveolar bone. In a superimposed view, the extraction socket was divided into three regions of equal size from the crestal-most bony top to the apex of the tooth: the coronal, middle, and apical regions. Dimensional alterations were calculated by subtracting the alveolar bone area in experimental sites from that of pristine tooth sites in each region. The vertical distance was measured from the crestal-most reference line that passed perpendicularly along the long axis of tooth to the outer surface of resorbed alveolar bone.

#### 2.3.4. Histologic/Histomorphometric Analysis

Specimens were decalcified in 5% formic acid, and embedded in paraffin. Serial sections (5 μm-thickness) were cut coronally along the center of the augmented sinus and in the buccolingual plane from the center of the extraction sockets. The two central-most sections were chosen and stained with Masson’s trichrome. The histologic slides were observed and digitally captured using a light microscope (BX50, Olympus, Tokyo, Japan). Histomorphometric measurements were made using a PC-based image-analysis system (Image-Pro Plus, Media Cybernetics). The quality of the grafted area from each histologic specimen of both experiments was evaluated by quantifying the relative composition of the total augmented area (%) with respect to newly formed bone (NB), residual material (RM), adipose tissue (AT), blood vessel (BV), and connective tissue (CT).

### 2.4. Statistical Analysis

The results of all experiments were analyzed using SPSS software (version 19.0, SPSS, Chicago, IL, USA). The mean and standard deviation (SD) were calculated, and all data were presented as mean ± SD values. All *in vitro* experiments were performed in triplicate, and one-way analysis of variance (ANOVA) was used for comparison between experimental groups with the same preincubation time. In an *in vivo* experiment of rabbit sinus augmentation, one-way ANOVA was used to compare the groups in the same observation period, and unpaired *t*-test was performed to analyze the differences between two groups with the same material but different observation periods. Repeated-measures ANOVA followed by post hoc Scheffé’s comparison was used to analyze the experimental extraction-socket grafting in dogs. The threshold for statistical significance was set at *p* = 0.05.

## 3. Results

### 3.1. In Vitro Cell Differentiation Induced by rhBMP-2 Released from Its Carrier System

**Figure 1:**
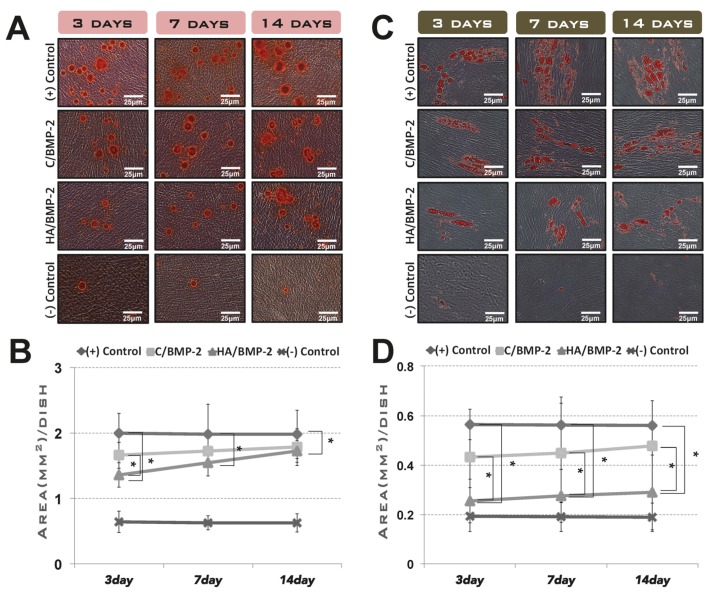
*In vitro* assay to explore the cell differentiation induced by rhBMP-2 released from two carrier systems. (**A**) Mineralized nodules formed after four weeks of osteogenic induction and were stained with Alizarin Red (original magnification ×400, scale bar = 25 μm); (**B**) The total areas of mineralized nodules were morphometrically measured. The area of mineralized nodules was largest in positive control group, regardless of the preincubation period. There were statistically significant differences in the degree of osteogenic induction between the positive control group and the HA/BMP-2 group at all preincubation periods (* *p* < 0.05); (**C**) Lipid vacuoles were observed by Oil Red O staining after four weeks of adipogenic induction (original magnification ×400, scale bar = 25 μm); (**D**) The total areas of lipid vacuoles were also measured. The degree of adipogenic induction was significantly lower in the HA/BMP-2 group than in both the positive control and C/BMP-2 groups (* *p* < 0.05). (+) Control, Positive control group; (−) Control, Negative control group; C, absorbable collagen sponge; BMP-2, bone morphogenetic protein-2; HA, hydroxyapatite-coated collagen.

Multiple mineralized nodules and lipid vacuoles were observed at all induced sites of the positive control group and both experimental groups with released rhBMP-2, although negative control sites preincubated with standard medium also exhibited limited formation of these structures. The area of mineralized nodules and lipid vacuoles was largest at positive control sites preincubated with rhBMP-2 solution, uniformly regardless of the preincubation period (Figure 1). The C/BMP-2 and HA/BMP-2 groups exhibited decreased osteogenic and adipogenic induction compared to the positive control group, but the induction of cell differentiation increased with increasing preincubation periods (3, 7, and 14 days) and release of ErhBMP-2. There were statistically significant differences in osteogenic induction between positive control group and HA/BMP-2 group for all preincubation periods (Figure 1B), but adipogenic induction in the HA/BMP-2 group was significantly reduced compared to the both positive control and C/BMP-2 groups (Figure 1D).

### 3.2. In Vivo Tissue Regeneration in the Rabbit Sinus-Augmentation Model

#### 3.2.1. Radiographic Analysis

**Figure 2:**
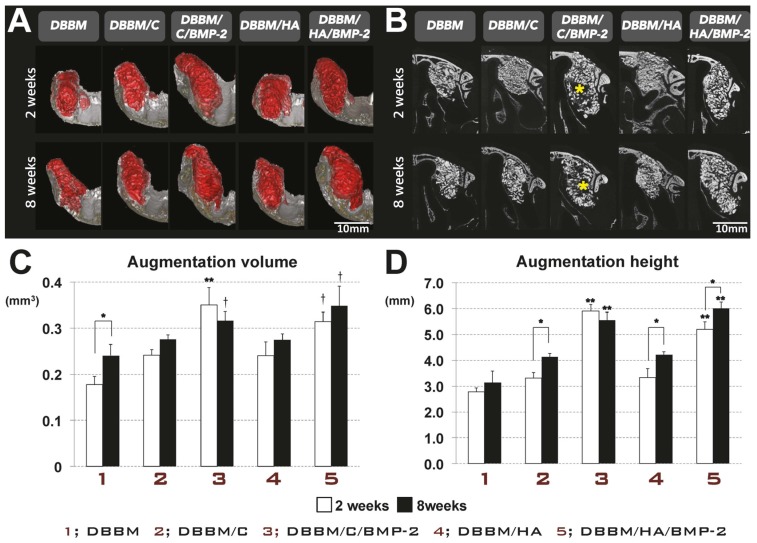
Radiographic analysis: micro-computed tomography in the rabbit sinus-augmentation model. (**A**) Three-dimensionally reconstructed views of the augmented sites at two weeks and eight weeks (scale bar = 10 mm); (**B**) Representative coronal cross-sectional images at two weeks and eight weeks (scale bar = 10 mm). Coronally sectioned views of the DBBM/C/BMP-2 group specifically exhibited radiolucent zones (* yellow) within the augmented area. These radiographic voids also decreased between weeks 2 and 8. Radiographically measured results demonstrated that the two experimental groups that received rhBMP-2 exhibited increased augmentation volume (AV; **C**) and augmentation height (AH; **D**) compared to the other three groups in which rhBMP-2 was not applied (** *p* < 0.05). The AV was significantly larger in both rhBMP-2-applied groups than in the DBBM group († *p* < 0.05). AH was measured linearly on the cross-sectional images. Regardless of the healing period, AH was significantly greater in the rhBMP-2-applied groups than in the other three groups (** *p* < 0.05). AH increased between weeks 2 and 8 in all groups except the DBBM/C/BMP-2 group (* *p* < 0.05). DBBM, deproteinized bovine bone mineral; C, absorbable collagen sponge; BMP-2, bone morphogenetic protein-2; HA, hydroxyapatite-coated collagen.

Both experimental sites (DBBM/C/BMP-2 and DBBM/HA/BMP-2) exhibited increased AV and AH compared to the other three groups (DBBM, DBBM/C, and DBBM/HA) (Figure 2). Regardless of the observation period (*i.e*., two or eight weeks), the AH and AV were significantly greater in both rhBMP-2-applied groups than in the DBBM group (*p* < 0.05). Sites that received DBBM and ACS or HA-coated collagen appeared to exhibit a greater AH and AV than sites that received DBBM only, which might be attributable to the additional grafted volume of the collagen. The differences, however, were not statistically significant. The AH and AV in the DBBM/C/BMP-2 group decreased from two to eight weeks, while conversely, those in all four of the other groups increased over time. Interestingly, coronally sectioned views of the DBBM/C/BMP-2 group specifically demonstrated radiolucent zones within the augmented area, and these radiographic voids also decreased in size between two and eight weeks.

#### 3.2.2. Histologic Analysis

**Figure 3:**
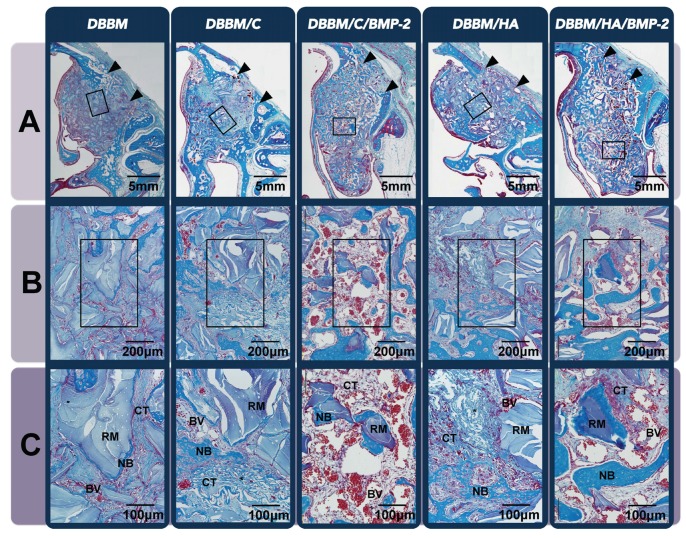
Histologic analysis in the rabbit sinus-augmentation model at the two-week healing point. (**A**) Low-magnification image of the entire sinus-augmentation site in each group after two weeks of healing (Masson’s trichrome staining, original magnification ×40, scale bar = 5 mm). Arrowheads indicate surgically created window sites. Newly formed bone (NB) could be observed all around the augmented area, but was concentrated locally around the grafted particles and near to the preexisting bone/Schneiderian membrane; (**B**) Representative high-magnification view (Masson’s trichrome staining, original magnification ×100, scale bar = 200 μm). (**C**) A twofold-enlarged images in the center of (**B**) (Masson’s trichrome staining, magnification, ×200, scale bar = 100 μm). A peculiar healing pattern was observed at both experimental sites in which rhBMP-2 was grafted; a very large amount of blood vessel (BV) formation can be seen in all areas between the particles and it was significantly higher in the DBBM/C/BMP-2 group than in the DBBM/HA/BMP-2 group. Arrowheads, surgically created entrance of window opening; DBBM, deproteinized bovine bone mineral; C, absorbable collagen sponge; BMP-2, bone morphogenetic protein-2; HA, hydroxyapatite-coated collagen; RM, residual material; AT, adipose tissue; CT, connective tissue.

In histologic samples of the two-week groups, NB could be observed all around the augmented area, especially around the grafted particles and nearby preexisting bone/Schneiderian membrane (Figure 3A). Remarkable new bone formation in the area near to the Schneiderian membrane was a representative feature of both experimental groups; DBBM/C/BMP-2 and DBBM/HA/BMP-2. However, the overall proportions of NB in the total augmented area were comparable regardless of the grafted biomaterials or the use of rhBMP-2 (Table 1).

**Table 1:** Histomorphometric results in sinus-augmentation model: proportion of composition in the total augmented area (mean ± SD values).

Group	DBBM		DBBM/C		DBBM/C/BMP-2		DBBM/H		DBBM/H/BMP-2	
	2 weeks	8 weeks	2 weeks	8 weeks	2 weeks	8 weeks	2 weeks	8 weeks	2 weeks	8 weeks
NB (%)	10.42 ± 0.43	25.29 ± 4.16 *	10.58 ± 2.30	24.87 ± 4.69 *	12.09 ± 2.14	28.42 ± 2.51 *	10.97 ± 2.04	23.01 ± 3.50 *	13.29 ± 6.02	30.80 ± 5.43 *
RM (%)	29.10 ± 4.33	19.71 ± 3.89	17.52 ± 2.01	16.09 ± 3.01	13.27 ± 2.32	13.95 ± 3.13	18.68 ± 2.90	15.83 ± 3.63	15.26 ± 0.39	11.63 ± 1.05
AT (%)	0.15 ± 0.08	0.65 ± 0.61	0.18 ± 0.06	1.55 ± 1.00	0.12 ± 0.02	12.92 ± 2.91 *^†§^	0.21 ± 0.10	1.88 ± 0.92	0.11 ± 0.03	5.96 ± 2.05 *^#^
BV (%)	1.37 ± 0.54	2.42 ± 1.04	1.45 ± 0.53	3.04 ± 1.30	6.26 ± 1.73 *^†§^	2.83 ± 0.50	1.34 ± 0.48	3.10 ± 1.58	3.87 ± 0.77 ^†^	2.48 ± 0.50
CT (%)	58.96 ± 4.15	51.93 ± 7.17	70.28 ± 3.04	54.46 ± 4.11	68.25 ± 3.63 *^#^	41.88 ± 5.22	68.80 ± 3.86	56.19 ± 2.71	67.47 ± 6.29 ^#^	49.13 ± 9.00

* Significantly different between 2 and 8 weeks (*p* < 0.05); † Significantly different from BMP-2 untreated group (*p* < 0.05); # Significantly different from DBBMgroup (*p* < 0.05); § Significantly different from DBBM/H/BMP-2 group (*p* < 0.05); NB, new formed bone; RM, residual material; AT, adipose tissue; BV, blood vessel; CT, connective tissue.

**Figure 4:**
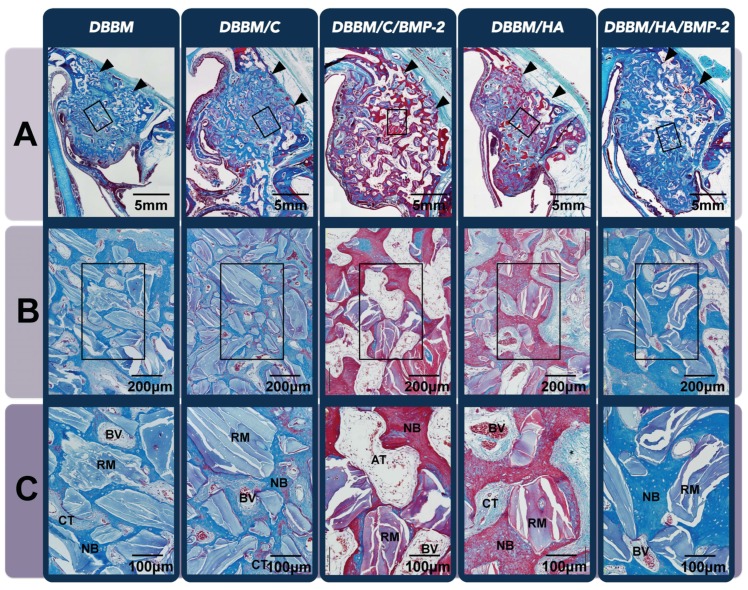
Histologic analysis in the rabbit sinus-augmentation model at the eight-week healing point. (**A**) Low-magnification image of the entire sinus-augmentation site in each groups after eight weeks of healing (Masson’s trichrome staining, original magnification ×40, scale bar = 5 mm). Newly formed bone (NB) was evenly distributed at all of the grafted area regardless of the experimental group, and appeared as a more mature type with increased lamellar bone compared to the sites in the two-week groups; (**B**) Representative high-magnification view (Masson’s trichrome staining, original magnification ×100, scale bar = 200μm); (**C**) A twofold-enlarged image in the center of (**B**) (Masson’s trichrome staining, magnification ×200, scale bar = 100μm). There was a very large amount of adipose tissue (AT) formation within the grafted area in both samples that received rhBMP-2, and it was significantly higher in the DBBM/C/BMP-2 group than in the DBBM/HA/BMP-2 group. Samples of the other three groups showed dense connective tissues (CT) in the area between the residual material (RM) and NB. Reduction of AT formation when using an HA-coated collagen as a carrier for BMP-2 enhances the bone quality. Arrowheads, surgically created entrance of window opening; DBBM, deproteinized bovine bone mineral; C, absorbable collagen sponge; BMP-2, bone morphogenetic protein-2; HA, hydroxyapatite-coated collagen; BV, blood vessel.

Another peculiar healing pattern was observed at both experimental sites that were grafted with ErhBMP-2—A very large amount of BV was formed in spaces between the particles (Figure 3B,C). This feature was not found in the other three groups (*i.e*., DBBM, DBBM/C and DBBM/HA). Histomorphometrically, both sites received ErhBMP-2 exhibited a statistically significant increase in proportion of BV compared to the other three groups at 2 weeks, and it was significantly higher in the DBBM/C/BMP-2 group than in the DBBM/HA/BMP-2 group (Table 1). However, this pattern was decreased in all samples of both groups at eight weeks. Instead, another specific feature within the grafted area of both samples that received ErhBMP-2 was a significantly increased amount of AT formation, although samples of the other three groups exhibited dense CT (Figure 4). DBBM/C/BMP-2 sites showed significantly greater AT formation than DBBM/HA/BMP-2. NB was evenly distributed in all experimental/control grafted areas, and appeared as a more mature type with increased lamellar bone compared to the sites of the two-week groups (Table 1).

### 3.3. In Vivo Tissue Regeneration in the Dog Extraction-Socket Model

#### 3.3.1. Radiographic Analysis

**Figure 5:**
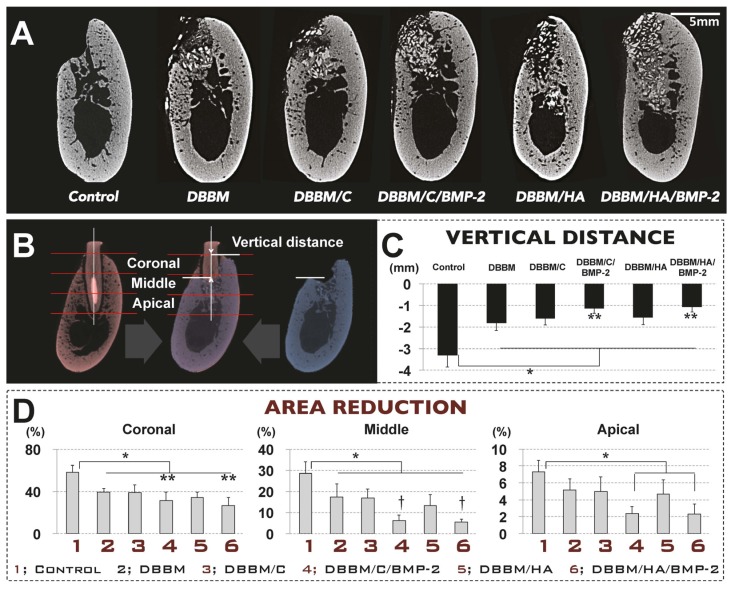
Radiographic analysis: micro-computed tomography of the beagle extraction-socket model. (**A**) Buccolingual cross-sectional view of the augmented sites after eight weeks of healing (scale bar = 5mm). The alveolar ridge resorption was more pronounced in the buccal and coronal regions than in the lingual and apical regions; (**B**) Schematic indicating the locations of the various measurements, using superimposing central-most sagittal views of experimental site (extracted and/or grafted) and pristine tooth site at the contralateral mandible; (**C**) The reduction of alveolar bone height in the fresh extraction socket was significantly decreased in all of the grafted groups compared to the control group (* *p* < 0.05). Two augmentation sites that received rhBMP-2 showed a significantly decreased vertical distance compared to the other three grafted groups (** *p* < 0.05); (**D**) All grafted groups showed decreases in the reduction of the alveolar bone area compared to the control group (* *p* < 0.05). These dimensional reductions of the alveolar ridge at sites that received rhBMP-2 were also significantly decreased than in the other grafted extraction-socket groups (**, significantly different from groups not treated with rhBMP-2; †, significantly different from the DBBM and DBBM/C groups). DBBM, deproteinized bovine bone mineral; C, absorbable collagen sponge; BMP-2, bone morphogenetic protein-2; HA, hydroxyapatite-coated collagen.

Alveolar ridge resorption following tooth extraction was observed in all groups, and was more pronounced in buccal and coronal regions than in the lingual and apical regions (Figure 5A). However, the reduction of the alveolar bone area and height in the fresh extraction socket was significantly decreased in all of the grafted groups compared to the control group (Figure 5C). In addition, dimensional alterations of alveolar ridge in DBBM/C/BMP-2 and DBBM/HA/BMP-2 groups were also significantly less than in the other grafted extraction-socket groups (*i.e*., the DBBM, DBBM/C and DBBM/HA groups) (Figure 5D).

#### 3.3.2. Histologic Analysis

**Figure 6:**
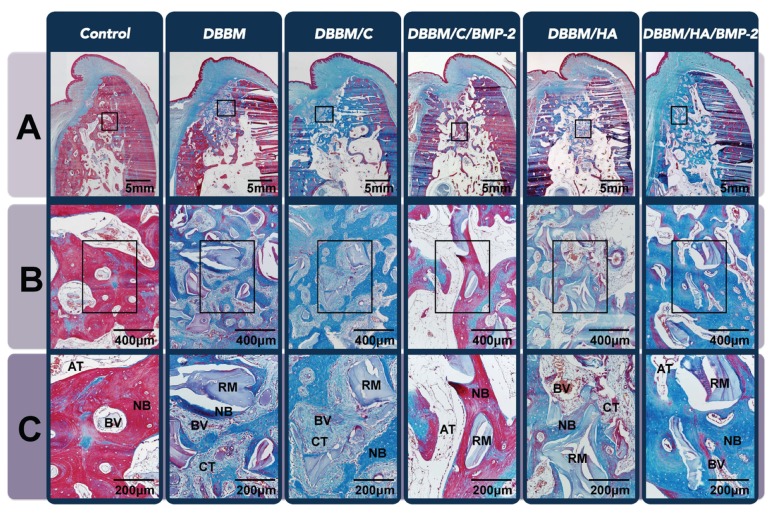
Histologic analysis of the beagle extraction-socket model. (**A**) Low-magnification image of the extraction site after eight weeks of healing (Masson’s trichrome staining, original magnification ×40, scale bar = 5 mm). While control sites showed a significant decrease in alveolar bone width at the coronal half of the dental root length, buccal bone plates at the other five grafted sites were in a higher position than at the control site; (**B**) Representative high-magnification view of the coronal region of the augmented area (Masson’s trichrome staining, original magnification ×100, scale bar = 400 μm); (**C**) A twofold-enlarged image in the center of (**B**) (Masson’s trichrome staining, magnification ×200, scale bar = 200 μm). Sites that received rhBMP-2 appeared to be in a more mature healing phase and showed higher proportions of adipose bone marrow tissue compared to the other three grafted sites, in which dense connective tissue could be found instead of adipose bone marrow tissue. DBBM, deproteinized bovine bone mineral; C, absorbable collagen sponge; BMP-2, bone morphogenetic protein-2; HA, hydroxyapatite-coated collagen; NB, newly formed bone; RM, residual material; AT, adipose tissue; BV, blood vessel; CT, connective tissue.

All sites healed with buccal bone plate resorption, but the histologic amounts of dimensional alteration differed between the groups according to the graft materials used. Alveolar bone area in coronal half of the dental root length at control sites was significantly lower than the other experimental sites (Figure 6A). However, the DBBM/C/BMP-2 and DBBM/HA/BMP-2 sites exhibited significantly higher proportions of adipose bone marrow tissue compared to the other three grafted sites (*i.e*., the DBBM, DBBM/C and DBBM/HA), in which dense CT could be found instead of adipose bone marrow tissue (Figure 6B,C). Interestingly, DBBM/C/BMP-2 group had a significantly higher proportion of adipose bone marrow tissue than the DBBM/HA/BMP-2 group, even though there were similar proportions of that tissue in DBBM/C and DBBM/HA groups (Table 2).

**Table 2:** Histomorphometric results in extraction-socket grafting model: proportion of composition of the augmented area (mean ± SD values).

Group	Control	DBBM	DBBM/C	DBBM/C/BMP-2	DBBM/H	DBBM/H/BMP-2
NB (%)	39.72 ± 12.85	31.58 ±5.92	28.44 ± 8.37	42.26 ± 8.46	26.11 ± 4.93	52.92 ± 7.33 ^†^
RM (%)	0.00	19.78 ± 3.50	20.22 ± 8.13	8.96 ± 3.93 ^†^	17.73 ± 5.81	4.71 ± 1.19 ^†^
AT (%)	26.07 ± 8.08 ^†^	10.19 ± 1.20	12.27 ± 0.88	27.20 ± 8.43 ^†§^	12.70 ± 2.31	16.16 ± 6.12 ^†^
BV (%)	6.88 ± 2.43 ^†§^	2.74 ± 1.07	1.60 ± 0.91	4.46 ± 1.24	3.24 ± 0.59	3.61 ± 2.04
CT (%)	27.33 ± 5.69	35.71 ± 8.99	37.47 ± 8.86	17.13 ± 5.61 ^†^	40.23 ± 6.68 ^#§^	22.60 ± 6.29 ^†^

† Significantly different from BMP-2 untreated group (*p* < 0.05); # Significantly different from DBBM/C/BMP-2group (*p* < 0.05); § Significantly different from DBBM/H/BMP-2 group (*p* < 0.05); NB, new formed bone; RM, residual material; AT, adipose tissue; BV, blood vessel; CT, connective tissue.

## 4. Discussion

This study was based on the hypothesis that HA-coating on collagen carrier would influence the direction of cellular differentiation due to its feature of controlled-release of rhBMP-2. Therefore, two types of cellular differentiation (osteogenic and adipogenic differentiation) were included in the *in vitro* test, because these are the representative decisive factors for bone quality. In the present study, cells grown in medium to which ErhBMP-2 was added directly exhibited the highest level of both osteogenic and adipogenic differentiation. However, cells in C/BMP-2 and HA/BMP-2 groups demonstrated significantly reduced formation of mineralized nodules and lipid vacuoles, and especially cells in the HA/BMP-2 group, which had the significantly lowest values of osteogenesis and adipogenesis in the initial release period (which was 3 days in this study). HA-coating onto ACS reduced initial burst release of rhBMP-2, and showed controlled profile of rhBMP-2 release with the increase of the proportion of HA coating in our previous study: [14] 98% and 40% of the loaded rhBMP-2 were released from the collagen and HA-coated collagen for 3 days. In addition, another one of our previous studies showed that controlled release of rhBMP-2 reduced adipose tissue formation of unwanted tissue response by rhBMP-2 and enhanced bone quality [11]. Many previous studies reported clinically negative effects of rhBMP-2 on bone density by adipose tissue formation [9,10,11,12], and the present results also demonstrated formation of rhBMP-2-specific tissues (vessels and adipose tissue) in sites that received ErhBMP-2. Osteogenesis and adipogenesis gradually increased with increase of preincubation time in both experimental releasing groups. These are in accordance with many previous studies showing increased cellular differentiation with increased concentrations of rhBMP-2 [17,18], and decreased *in vivo* AT formation by other controlled-releasing carrier systems [19,20].

A particularly interesting finding in this result was that the controlled release of rhBMP-2 reduced adipogenic differentiation significantly more than osteogenic differentiation (Figure 1). Reduction in release of ErhBMP-2 may affect adipose formation more than mineralized tissue formation at the *in vitro* level, suggesting that control of the initial burst release from the carrier might reduce AT formation and enhance bone density. These findings are consistent with the present *in vivo* experimental results and the results from previous studies [12]. The reduction in the initial burst concentration by controlled-releasing carrier systems was shown to enhance bone density and reduce AT formation [11]; on the other hand, the increase in rhBMP-2 concentration induced seroma formation [21,22] or cystic changes [12,23]. Although adipogenic and osteogenic differentiation by BMP-2 are dependent upon different binding receptors and target molecules, it is interesting to note that two types of BMP pathway share the same downstream signaling events: the Smad and p38 mitogen-activated protein kinase (MAPK) pathways. These *in vitro* and *in vivo* results suggest that a high concentration of rhBMP-2 move those biologic pathways toward adipogenic differentiation, although little is known about that mechanism.

The present *in vivo* results for the augmented sinus also revealed a peculiar healing pattern at two weeks after surgery: the overwhelming formation of newly formed vessels despite limited sources for angiogenesis. This feature has not been reported for any defect or ectopic models *in vivo*. The sinus-augmentation model is a type of contained defect that is surrounded by limited healing sources (e.g., the Schneiderian membrane) that contain minimal cells and glandular tissues. This environment could have provided a low-oxygen state within the grafted area filled with biomaterials and blood coagulum in the early healing phase. This hypoxic state might regulate the ErhBMP-2 activity from the undifferentiated to endothelial cells [24]. However, this particular healing pattern had disappeared at 8 weeks, and increased AT formation could be observed at both experimental sites that received rhBMP-2 compared to all non-rhBMP-2 sites, which were filled with fibrovascular connective tissues instead of AT. However, these specific tissues (BV and AT in the early and late healing phase, respectively) were significantly reduced in the sinuses of DBBM/HA/BMP-2, which could be supported by the present *in vitro* results. However, the underlying mechanism and the relationship between angiogenesis and adipogenesis require further study.

An enhanced bone density (and reduced AT formation) of the regenerated tissue was also observed in the extraction-socket model that received rhBMP-2-loaded HA-coated collagen. In addition, osteoinductive activity of ErhBMP-2 could decrease the buccolingual shrinkage of the alveolar ridge following tooth extraction. Many preclinical and clinical studies have found significant alteration of the alveolar ridge dimensions in the coronal area of the extraction socket [25,26]. Araujö *et al.* found a 35% reduction in the buccolingual dimension of the coronal area after extraction, but minimal alterations in both the middle and apical areas were noted [25]. However, more vigorous reductions of the alveolar ridges in the coronal (58%) and middle (29%) areas were observed in control sites of the present study. This finding may be attributable to the difference between the experimental models used: Araujö *et al.* used a single root extraction socket, while a multiple extraction-socket model was used in the present study. Interestingly, unlike control sites, experimental sites that received ErhBMP-2—regardless of the carrier system—exhibited well-maintained grafted biomaterials beyond the resorbed buccal bone level. Osteoinductivity by rhBMP-2 might cause mineralized tissue formation in the area, as has already been shown in other ectopic models [10,27,28,29]. Both quantitatively and qualitatively, ErhBMP-2-loaded HA-coated collagen may be beneficial for extraction-socket treatment, even at sites with multiple extractions. This may be produced by enhancement of bone formation rather than bone remodeling via osteoclast activation; however, further study is needed to confirm this suggestion.

While two contained-types of *in vivo* models were used in the present study (the sinus-augmentation and extraction-socket models), there are some differences between them; including the types of cells present, the relative abundance (or lack there of) of healing sources, and the clinical goal of treatment. Researchers and clinicians would expect the application of BMP-2 to accelerate the bone healing processes in the sinus-augmentation model and reduce the healing period, and reduce the dimensional alterations of alveolar ridge in the extraction-socket model. Therefore, different observational periods and measurement parameters were used discriminately in each model. In the grafted sinuses, a peculiar healing pattern and inferior bone density was observed in the early healing period, and ErhBMP-2 failed to shorten healing time despite reduction of complication by HA-coated collagen. However, the extraction socket healed with bony augmentation at sites that received ErhBMP-2, and its dimensions were maintained. In addition, HA-coated collagen carriers enhanced bone regeneration qualitatively and quantitatively in the socket model.

## 5. Conclusions

In conclusion, HA-coated collagen carriers for ErhBMP-2 may reduce *in vitro* induction of adipogenic differentiation and *in vivo* adipose bone marrow tissue formation in bone tissue engineering by ErhBMP-2. These results suggest that HA-coated collagen carrier systems loaded with ErhBMP-2 may reduce adipose tissue formation, and can ultimately be expected to enhance clinical bone quality in rhBMP-2-engineered tissues. ErhBMP-2 failed to accelerate the bone-healing processes in the sinus-augmentation model, despite the use of HA-coated collagen as a carrier. However, rhBMP-2-loaded HA-coated collagen could be a candidate for extraction-socket therapy, as evidenced by the quantitative and qualitative enhancement of bone regeneration in the socket-grafting model.
